# Assessment of chiropractic treatment for active duty, U.S. military personnel with low back pain: study protocol for a randomized controlled trial

**DOI:** 10.1186/s13063-016-1193-8

**Published:** 2016-02-09

**Authors:** Christine M. Goertz, Cynthia R. Long, Robert D. Vining, Katherine A. Pohlman, Bridget Kane, Lance Corber, Joan Walter, Ian Coulter

**Affiliations:** Palmer College of Chiropractic, Palmer Center for Chiropractic Research, 741 Brady Street, Davenport, IA 52803 USA; RAND Corporation, 1776 Main Street, Santa Monica, CA 90401 USA; Samueli Institute, 1737 King Street, Suite 600, Alexandria, VA 22314 USA; Research Institute, Parker University, 2540 Walnut Hill Lane, Dallas, TX 75229 USA

**Keywords:** Low back pain, Chiropractic, Comparative effectiveness, Military, Spinal manipulative therapy, Pragmatic clinical trial

## Abstract

**Background:**

Low back pain is highly prevalent and one of the most common causes of disability in U.S. armed forces personnel. Currently, no single therapeutic method has been established as a gold standard treatment for this increasingly prevalent condition. One commonly used treatment, which has demonstrated consistent positive outcomes in terms of pain and function within a civilian population is spinal manipulative therapy provided by doctors of chiropractic. Chiropractic care, delivered within a multidisciplinary framework in military healthcare settings, has the potential to help improve clinical outcomes for military personnel with low back pain. However, its effectiveness in a military setting has not been well established. The primary objective of this study is to evaluate changes in pain and disability in active duty service members with low back pain who are allocated to receive usual medical care plus chiropractic care versus treatment with usual medical care alone.

**Methods/design:**

This pragmatic comparative effectiveness trial will enroll 750 active duty service members with low back pain at three military treatment facilities within the United States (250 from each site) who will be allocated to receive usual medical care plus chiropractic care or usual medical care alone for 6 weeks. Primary outcomes will include the numerical rating scale for pain intensity and the Roland-Morris Disability Questionnaire at week 6. Patient reported outcomes of pain, disability, bothersomeness, and back pain function will be collected at 2, 4, 6, and 12 weeks from allocation.

**Discussion:**

Because low back pain is one of the leading causes of disability among U.S. military personnel, it is important to find pragmatic and conservative treatments that will treat low back pain and preserve low back function so that military readiness is maintained. Thus, it is important to evaluate the effects of the addition of chiropractic care to usual medical care on low back pain and disability.

**Trial registration:**

The trial discussed in this article was registered in ClinicalTrials.gov with the NCT01692275 Date of registration: 6 September 2012

## Background

Low back pain (LBP) is well recognized as a prevalent and burdensome health problem in both military and civilian populations [1, 2]. It is also one of the most common reasons why members of the U.S. armed forces seek medical care [3, 4]. LBP, common in both deployed and non-deployed military personnel [5], is also among the most likely conditions to interrupt combat duty [2, 6]. In army personnel, LBP represents the highest 5-year risk factor for permanent disability [7].

Because of the combined costs associated with personal suffering, healthcare, and disability expenditures, and the resulting impaired capacity of personnel to conduct military operations, LBP has been characterized as “the silent military threat” [8, 9]. Development of a more effective, early treatment that prevents chronicity and reduces recurrence is likely to mitigate some of the deleterious effects of LBP on individuals and the military healthcare system.

In the United States, the chiropractic profession contains more than 70,000 actively licensed practitioners [10] who specialize in conservative treatment for musculoskeletal conditions with a special focus on spinal health [11]. At least 7.5 % of the U.S. population seeks chiropractic care each year, representing over 190 million patient visits annually [12, 13]. The care offered by doctors of chiropractic (DCs) is consistently rated highly by patients in studies assessing satisfaction [14–17]. Randomized controlled trials (RCTs) have demonstrated that chiropractic care and its signature treatment, spinal manipulation, is an effective conservative care option for patients with LBP [18–21]. Chiropractic care or spinal manipulation is also endorsed as an evidence-based, cost effective, conservative treatment option in the clinical practice guidelines for patients with acute, subacute, and chronic LBP [22–24].

DCs provide care in private practice and in multidisciplinary healthcare settings, including Veterans Affairs and military health treatment facilities [25, 26]. Currently, chiropractic care is available at 65 military health treatment facilities within the United States and internationally [27].

Goertz et al. conducted a pilot RCT comparing the effectiveness of chiropractic care plus standard medical care with standard medical care alone for active duty military personnel with acute LBP [28]. This study reported clinically and statistically significant greater improvement in pain and disability in the group including chiropractic care. However, the study was conducted at a single military installation with a relatively small sample (n = 91). This paper describes a larger scale, multisite, comparative effectiveness study at three geographically and demographically diverse U.S. military medical treatment facilities. Because chiropractic care for LBP in the military is delivered within a multidisciplinary framework of care, rather than as a single system of care, the study is focused on the comparative effectiveness of chiropractic care plus usual medical care with usual medical care alone, in a pragmatic design.

### Specific aims

The primary aim of this pragmatic comparative effectiveness study is to compare pain and disability of active duty military personnel with LBP who are treated with chiropractic care and usual medical care compared with those treated with usual medical care alone. We hypothesize that those allocated to receive both chiropractic care and usual medical care will show greater reduction in pain and disability than those receiving usual medical care alone.

Secondary aims explore the effects of adding chiropractic care to usual medical care on healthcare utilization, medication use, and quality of life.

## Methods

### Overview

The **A**ssessment of **C**hiropractic **T**reatment for LBP in Active Duty Military Personnel (ACT 1) is a pragmatic, prospective, multisite, parallel group comparative effectiveness study with adaptive allocation [29–31]. ACT 1 is being conducted at Naval Medical Center San Diego, California (NMCSD), Naval Hospital Pensacola, Florida (NHP), and Walter Reed National Military Medical Center (WRNMMC), Bethesda, Maryland. Two hundred and fifty participants with chronic, subacute, or acute non-surgical LBP are being enrolled at each site (total of 750).

**Fig. 1:**
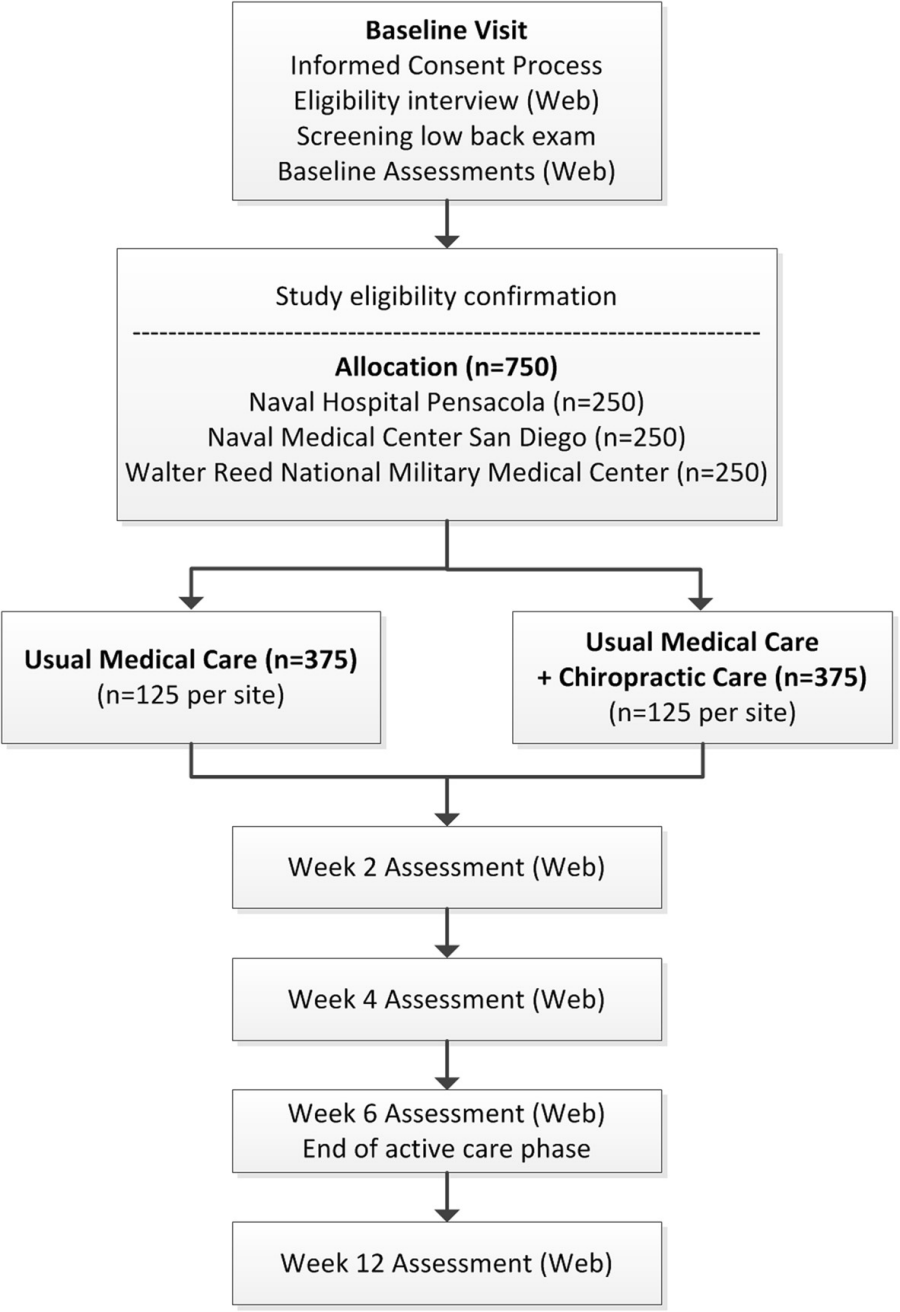
ACT 1 study flow chart

Participants meeting eligibility criteria are allocated to one of two treatment groups: usual medical care (UMC) plus chiropractic care and UMC alone. The active care phase of the study is 6 weeks from group allocation. Patient-reported outcomes (PROs) are assessed at baseline (prior to randomization) and at 2, 4, 6, and 12 weeks from allocation with the primary endpoint at 6 weeks (Fig. 1).

The trial is managed through the Submission Tracking and Reporting System (STaRS), a comprehensive web application developed by the Palmer Center for Chiropractic Research (PCCR) with a dual purpose of collecting outcome assessments for study participants and serving as a secure electronic data capture and clinical trial management system for study personnel. The STaRS application is available for participants to access 24 hours a day throughout the duration of the trial. Study staff use STaRS for data entry, confirmation of participant eligibility, and study event reporting. STaRS also provides real-time reports for study management.

### Trial organization

The research team comprises individuals from three collaborating institutions: the RAND Corporation, the PCCR, and the Samueli Institute. The RAND Corporation manages the financial aspects, overall administration, and Institutional Review Board (IRB) issues of the grant award, as well as required deliverables to the Department of Defense (DoD) program officer. The Samueli Institute advises on processes for conducting research within the military and ensures compliance with the entities that regulate the conduct of human subjects’ clinical research within the DoD to include the U.S. Army Medical Research and Material Command Human Research Protection Office and the Army’s Clinical Investigation Regulatory Office.

Investigators from the PCCR are responsible for developing, implementing, and managing the trial at each of the three sites. Each trial site includes an active duty United States Naval or Army medical officer serving as principal investigator (PI), one or two DCs, and one PCCR site Project Manager (PM) locally stationed at the military treatment facility (MTF). The PM is responsible for day-to-day trial implementation at the respective MTF including the conduct of recruitment activities, participant tracking, and communication. A lead PM oversees trial operations at all three sites, acts as a liaison between the sites and trial co-investigators, and ensures protocol adherence and fidelity across sites. A central trial clinician reviews and monitors all adverse events.

### Clinical sites

Participating clinical sites are MTFs that had an already established chiropractic program. DCs delivering patient care for trial participants are civilians who were contracted on a full-time basis by the DoD within the MTF 15–20 years prior to initiation of the study. Further information about each site is briefly described below.

#### Naval Hospital Pensacola (NHP)

NHP and its nearby associated naval branch clinics provide healthcare services to active duty military personnel in the Pensacola, Florida region. Chiropractic care is available to active duty personnel at the Naval Branch Health Clinic Naval Air Technical Training Command. Chiropractic services are part of the Sports Medicine and Rehabilitative Therapy Clinic and have been available since September 2003. A single DC provides care for trial participants at the Naval Air Technical Training Command branch clinic.

#### Walter Reed National Military Medical Center (WRNMMC)

WRNMMC, located in Bethesda, Maryland, is the largest U.S. military medical center, providing services to over 1 million beneficiaries per year [32]. The chiropractic clinic at WRNMMC is located within the Department of Orthopedics and Rehabilitation and provides care to active duty service members with musculoskeletal conditions including service members with combat-related injuries. WRNMMC established chiropractic services in 1998. Two DCs provide care to trial participants at this site.

#### Naval Medical Center San Diego (NMCSD)

NMCSD is a large military healthcare system located in coastal southern California serving U.S. military personnel stationed at several surrounding military bases. NMCSD provides chiropractic care for active duty personnel as a special service of the Physical Therapy Department. Chiropractic services have been available at NMCSD since 2003. A single DC provides care to trial participants within the branch clinic at North Island, on Naval Base Coronado.

### Regulatory approvals

The trial protocol received ethics approvals from the following five Institutional Review Boards: Palmer College of Chiropractic (#2010G137), RAND Corporation (#2010-0782), NMCSD (#NMCSD.2012.0022, IRB of record: Naval Medical Center San Diego, California), NHP (#NHPC.2012.0002, IRB of record: Naval Medical Center Portsmouth, Virginia), and WRNMMC (#369462, IRB of record: Walter Reed National Military Medical Center Bethesda, Maryland). The study protocol was also approved by the U.S. Army Medical Research and Material Command Human Research Protection Office and the Clinical Investigation Regulatory Office. All study investigators have completed training in the protection of human subjects as required by the respective collaborating institutions.

Prior to study commencement, the collaborating investigative institutions also established a Cooperative Research and Development Agreement (CRADA) with each of the three participating MTFs. Final approval of the CRADA occurred in June 2012 and was renewed in April 2015. A data sharing agreement and systems security verification, under the auspices of TRICARE Management Activity, were established between the MTFs and the ACT 1 collaborating institutions (RAND Corporation and PCCR).

### Recruitment procedures

Active duty participants aged 18–50 years (inclusive) reporting acute, subacute, or chronic LBP who are able to provide voluntary written informed consent are eligible for this trial. Participants are ineligible if they have knowledge of a pending absence through the 6-week active treatment phase. Such absences could include a planned leave, deployment, temporary duty assignment, or permanent change of station. Participants unwilling to be allocated to either intervention arm are also ineligible. A detailed description of the inclusion/exclusion criteria is summarized in Table 1.

**Table 1:** Eligibility criteria

Inclusion criteria	Rationale
Age ≥ 18 and ≤ 50	Age range of most active duty U.S. military personnel
Acute, subacute, or chronic low back pain	Low back pain commonly treated by primary care and chiropractic providers
Ability to provide voluntary written informed consent	Able to comprehend study details; able to make decisions without limitations or impairment
Active duty status at one of the three participating military treatment facilities	Chiropractic care available only to active duty personnel at U.S. military treatment facilities
Exclusion criteria	
LBP from a non-musculoskeletal source (pain from a visceral condition[s])	Care outside study scope needed; potential to confound health outcomes
Co-morbid pathology that may directly impact spinal pain	Care outside study scope needed; potential to confound health outcomes
Recent spinal fracture (within the last 8 weeks)	May influence ability to measure pain-related health outcomes; safety concern
Recent spinal surgery (within the last 12 weeks)	Potential to confound health outcomes due to natural history or from potential complications
Spinal or paraspinal tumor(s)	Care outside study scope needed
Spinal or paraspinal infection(s)	Care outside study scope needed
Spinal inflammatory arthropathy (rheumatoid arthritis, enteropathic spondyloarthropathy)	Potential to confound health outcomes
Contraindication(s) for spinal manipulation of the lumbar spine and pelvis (unstable spinal segments, cauda equina syndrome)	Care outside study scope needed
Pregnancy or plans to become pregnant within active treatment period	Potential to confound health outcomes
Diminished/altered mental capacity	May prohibit informed consent or compromise safety or compliance with study procedures
Use of spinal manipulative care for any reason within the past month	Prevent carryover effects from recent chiropractic care
Significant/severe osteoporosis	Potential to confound health outcomes; care outside study scope may be needed
Unwilling to provide phone and electronic contact information	Compromises ability to adhere to study protocol
Unable to confirm availability during the active treatment period due to known deployment, orders for a distant duty assignment, or other absence	Compromises ability to adhere to study protocol
Does not agree to be enrolled regardless of group assignment	Compromises ability to adhere to study protocol
Post-traumatic stress disorder diagnosis	Potential to confound health outcomes

Patients with LBP enter the military healthcare delivery system through multiple pathways. Thus, the investigative team identified department clinics likely to diagnose or manage patients with LBP within each MTF and requested their assistance with recruitment efforts. Command support (permission) was obtained prior to study recruitment, from each respective department, to post IRB-approved study advertisements and recruit study participants. At WRNMMC, command support was obtained from the internal medicine, physical therapy, neurosurgery, and physical medicine and rehabilitation departments. At NHP, command support was received from the Department of Family Medicine, the Department of Orthopedics, and three branch clinics: Naval Air Station Pensacola branch clinic, Naval Air Technical Training Center, and Corry Station. At NMCSD, command support was obtained from the Department of Orthopedics, Naval Branch Health Clinics Miramar, Naval Base San Diego and Coronado, and the NMCSD Military Health Center.

### Participant screening

Trial participants are recruited via either self-referral or referral from a healthcare provider. IRB-approved recruitment materials are placed in patient waiting rooms and other approved areas within each MTF. In either recruitment method, participants must meet all clinically related eligibility criteria determined at exam and general criteria confirmed by the PM during the baseline interview. Individuals who do not meet all study eligibility criteria are excluded. Responses to the baseline eligibility interview and eligibility criteria obtained from the examining clinician are entered and electronically stored in STaRS.

Interested participants meet with the PM, who initiates the informed consent process in a private setting. The PM reviews the informed consent document, study flow chart, and specific visit activities with the participant. Individuals have the opportunity to read the informed consent document and ask questions about participation. Those wishing to participate sign the written informed consent document. Following the consent process, a baseline interview is conducted to obtain basic demographic information to screen for non-clinically determined eligibility criteria. After the baseline interview is submitted, STaRS assigns each participant a unique study number. Eligible participants are also assigned a temporary password to complete online assessments.

Initially eligible participants undergo a clinical evaluation of their low back by the healthcare provider who manages the condition (that is, a neurologist, physiatrist, or internist), a primary care provider, or an Independent Duty Corpsman. During the evaluation, the provider renders a professional opinion regarding clinically determined eligibility criteria such as whether or not the LBP is related to the musculoskeletal system, the need for additional diagnostic testing, and the existence of conditions posing a contraindication to spinal manipulation (such as acute spinal fracture or cauda equina syndrome). Eligibility information obtained from the clinical evaluation is documented by the provider on a paper form and provided to the PM, who enters the information into STaRS to determine eligibility. Alternatively, the LBP evaluation may be performed prior to an individual’s meeting with a PM as part of the patient’s standard of care.

#### Baseline assessment

Prior to allocation, all eligible participants complete a baseline assessment consisting of demographic information, expectations of care, and a series of PRO questionnaires that measure current pain intensity, the impact of the current LBP on functional status and quality of life, and self-reported medication use for LBP. The baseline assessment is conducted on dedicated study computers.

Participants access the baseline assessment questions by logging into STaRS using their email address as their username and the temporary password assigned by STaRS. STaRS requires all participants to change their password upon initial login, which is used to complete future online assessments.

#### Allocation

Participants remaining eligible after completing: 1) the consent process, 2) baseline interview, 3) clinical evaluation, and 4) baseline assessment are allocated to a treatment group. Allocation occurs within STaRS via an adaptive computer-generated minimization algorithm programmed to balance group assignment on the factors of sex (M, F), age (18 to <30, 30 to 50), LBP duration (<1 month, 1–3months, >3 months), and baseline Numeric Rating Scale (0–5, 6–10) measurement (worse pain in past 24 hours). Participants are allocated to one of two groups: 1) UMC or 2) UMC plus chiropractic care. PMs, participants, and all study personnel are unable to influence the group assignment, and future allocations are concealed.

### Study interventions

#### Usual medical care

In this pragmatic trial, UMC includes any care recommended or prescribed by a non-chiropractic military healthcare provider for the purpose of managing/treating LBP. UMC may include education about a condition, self-management advice, and pharmacologic pain management. Physical therapy and referral to a pain clinic may also be prescribed as a component of UMC. UMC providers report prescription medication class, referrals, and/or self-care recommendations. Participants allocated to the UMC group are asked by study personnel to avoid receiving chiropractic care for 6 weeks unless otherwise directed by their healthcare provider.

#### Usual medical care plus chiropractic care

Participants allocated to this group continue with prescribed UMC as described above and also receive up to 12 chiropractic visits during the 6-week active care period. Chiropractic treatment frequency, duration, and procedures are determined individually based on the participant’s condition, response to care, scheduling availability, and other factors pertinent to the case.

The primary therapeutic procedures delivered by DCs for LBP are thrust or non-thrust spinal manipulation in the low back and adjacent regions [33]. Treatment decisions regarding manipulation type, location, and direction are based on the LBP diagnosis and concurrent diagnoses. Other factors that inform treatment decisions include patient preference, prior response to care (if known), the presence or absence of local tenderness, paraspinal muscle hypertonicity, spinal joint hypomobility, positions of relief and/or provocation, and imaging findings (for example, spinal curvatures, congenital anomalies). Other therapeutic procedures delivered by the DC may include rehabilitative exercise, manual manipulation of upper and lower extremity joints and other spinal regions, interferential current therapy, ultrasound therapy, cryotherapy, heat therapy, and manual myofascial therapies.

### Outcome measures

PROs are collected at 2, 4, 6, and 12 weeks from allocation (Table 2). The primary endpoint is at 6 weeks and the secondary endpoint is at 12 weeks.

**Table 2:** Data collection schedule

	Assessment time point				
Outcome measure	Baseline (+/− 3 days)	Week 2 (+/− 3 days)	Week 4 (+/− 3 days)	Week 6 (+/− 7 days)	Month 3 (+/− 7 days)
Demographics	X				
Numeric Rating Scale for pain intensity	X	X	X	X	X
Roland-Morris Disability Questionnaire	X	X	X	X	X
Back Pain Functional Scale	X	X	X	X	X
Bothersomeness of symptoms	X	X	X	X	X
PROMIS-29	X			X	X
Global improvement measure		X	X	X	
Healthcare utilization and medication use	X	X	X	X	X

#### Primary outcome measures

The co-primary outcome measures are the Numeric Rating Scale (NRS) for average pain intensity during the past week and the Roland-Morris Disability Questionnaire (RMDQ). The NRS has excellent metric properties, is commonly used in RCTs studying LBP [34, 35], and has been demonstrated as a valid and reliable measure [36]. Participants are asked to rate their average level of LBP during the past week on an ordinal 11-box scale (0 = no LBP; 10 = worst possible LBP). The RMDQ is a reliable and valid LBP-related disability assessment instrument commonly used in clinical research [37, 38]. Containing 24 questions, it is considered sensitive to disability-related changes in patients with LBP [39–41].

#### Secondary outcome measures

Secondary outcome measures include the NRS of the worst LBP intensity during the past 24 hours, the Back Pain Functional Scale, bothersomeness of symptoms, Patient Reported Outcomes Measurement Information System (PROMIS)-29 variables, medication use, and healthcare utilization.

The Back Pain Functional Scale is a 12-question survey assessing functional status. Each question is answered using a 6-point scale (0 = unable to perform activity and 5 = no difficulty), resulting in scores ranging from 0 to 60 where the higher scores are equal to better functional status [41]. Bothersomeness of symptoms associated with LBP is measured by asking the patient to rate the bothersomeness of LBP during the past week, measured on a 1 to 5 scale (1 = not at all bothersome and 5 = extremely bothersome) [42, 43].

The PROMIS-29 is a set of questions that measure depression, anxiety, physical function, pain interference, fatigue, sleep disturbance, and satisfaction in social roles [44]. The PROMIS-29 instrument contains 29 questions; 4 items from each primary domain plus a single pain intensity rating. This outcome instrument is administered at baseline and at weeks 6 and 12 [45–47]. Perceived global LBP improvement is assessed using a question adapted from a study investigating the effect of expectations on patients with LBP [48]. Participants are asked to rate their perceived LBP improvement on a 7-point scale (0 = completely gone to 6 = much worse) at weeks 2, 4, and 6.

Participants are asked to indicate the type of healthcare providers who have treated their current episode of LBP and indicate how often they took pain relieving medication (both prescription and/or over-the-counter) during the past week. Choices are 0 days, 1–2 days, 3–4 days, 5–6 days, or 7 days.

### Data collection and management

#### Data collection

This trial uses paper data collection forms, electronic data capture through STaRS, and data abstracted from the participant’s electronic medical record.

The STaRS home page provides all users the same login that, upon validation, directs them to the appropriate section of the application according to login credentials. PMs enter baseline data, including the exam screening form, into customized logic-based electronic forms that provide validation checks to ensure participant eligibility prior to allocation.

Electronic data capture is used to collect PROs at 2, 4, 6, and 12 weeks from allocation (Table 2). Participants are directed to the outcome assessments. Many features were implemented to provide participants with a self-managed experience while collecting study data at respective intervals over the 12-week study period. Participants may complete the assessment from any device capable of supporting an internet connection and web browser. If a participant forgets or would like to change their password, a link is provided that sends them a temporary password to their email address. At the time of the next login with the temporary password, STaRS will prompt the user to define a new password for future use. Within the STaRS application, participants may also update their contact information at any time.

Outcome measures are collected in a linear manner across all time points. Primary outcome measures are required variables and must be completed as a whole, whereas secondary outcome measures may be skipped by the participant. Programmatic review prompts the users after each assessment for any missing variables and asks them to review and complete them before moving onto the next measure. A visual progress bar is provided at the bottom of the page to inform users of overall percent completed.

STaRS sends an email at pre-programmed intervals to remind participants to complete the online assessments. Outcome assessments are available to complete for a window of 6 days for weeks 2 and 4, and 14 days for weeks 6 and 12. Automated emails are programmed to be sent the day the window opens for the respective time point. An additional email will be sent by STaRS if the participant has not completed the assessment by the actual due date. To augment STaRS automated emails, the PM personally contacts each participant by text message, email, or telephone during the window for each assessment. Participants can inform the site PM if they are unable to access STaRS or complete the assessment for various reasons. All contact with participants is documented by the site PM within STaRS.

If a participant does not complete an assessment within the designated window (Table 2), a PM will attempt to collect the primary outcome measures using a computer-assisted telephone interview. A PM will attempt to collect the week 2 and 4 assessments within 3 days, and the week 6 and week 12 assessments within 7 days of the assessment expiration dates.

For participants in the group that also receive chiropractic care, the DC completes a paper data collection form for each study visit that details the type of spinal manipulation performed including the anatomical region, and other therapeutic procedures used with the corresponding diagnosis (ICD-9) and procedural (CPT) codes. The PM carefully tracks the number of study visits per patient that occur in the 6-week period and manages documentation for study visits. During the active care phase of the trial, PMs enter data from the study treatment forms into customized electronic forms. Data entry errors and change requests are submitted through a module within STaRS that provides an audit trail of who altered specific data elements and when they were altered.

To document UMC received for LBP and to explore healthcare utilization for LBP, information from healthcare provider visits for LBP that occur during the 12-week study period is abstracted from the participant’s electronic medical record. Data abstracted includes reason for visit, provider type(s), diagnoses, procedures conducted, and prescribed medications.

The dual purpose of the STaRS application allows for trial management tools. STaRS is programmed to produce reports to allow study staff to monitor participants through all phases of the study. Specific reports available to study staff include a screening report, which provides the number of participants screened as well as the reasons for exclusion, and a tracking report, which allows the user to view individual participant information such as date of important study visits, allocation, and the status of each outcome assessment. PMs can monitor trends with respect to missed outcomes. PMs also enter adverse events and protocol deviations into STaRS, which is programmed to provide emails to specific study investigators as well as research staff. This feature allows for central investigator oversight, which is especially important given the multiple site locations.

#### Data management and security

The STaRS application is 21 CFR part 11 compliant and integrated with a Central Participant Database and a Project/Users Permissions System to control project personnel access to web modules. PCCR registered the backtoaction.org site secured with Certified Secure Socket Layers (SSL) 128-bit encryption, hosted (IIS V6.0), and maintained by Palmer College of Chiropractic Information Services department. The web programmer developed the application in ASP.NET v4.0 in C# and Structured Query Language (SQL) using Microsoft Visual Studio 2010. All data are stored on an internal Microsoft SQL Server 2014. Only select study personnel have access to data via Microsoft SQL Server Management Studio 2014. All PCCR servers reside behind a stateful firewall with permissions determined by Active Directory.

The data core manager will perform a soft lock of the database (Microsoft SQL Server) and write programs in SAS System for Windows (Release 9.3) using SAS ACCESS in order to perform data cleaning procedures of range and consistency checks. Once all data edits are recorded and performed, the data core manager will coordinate with the programmer to perform a final lock removing all access to the database to ensure that no further changes to the data can be made. Final analyzable dataset(s) and the data dictionary will be created from the final locked database.

### Statistical methods

The data will be analyzed using an intention-to-treat approach in which participants will be analyzed according to their original treatment allocation. All observed data will be used in the analyses. Data analyses will be performed using SAS/STAT (Release 9.3) (SAS Institute Inc., Cary, NC).

#### Primary data analysis and sample size

The co-primary outcome variables (RMDQ and the NRS for LBP intensity) will be modeled with linear mixed effects regression over baseline and weeks 2, 4, 6, and 12. We will assume group means are the same at baseline, and include terms in the model for time (as a categorical variable), site, site-by-group, time-by-group, and site-by-time-by-group interactions, and the variables in the minimization algorithm. The covariance structure will be chosen by comparing the maximized log-likelihoods and the Bayesian information criteria for several covariance pattern models against the unstructured covariance. Diagnostics of the conditional predicted values and conditional residuals will be used to assess the assumption of normality and fit for each model.

The main results will be based on the final models for the co-primary outcome variables at the end of the active care phase (6 weeks). If the site-by-time-by-group interaction is significant at the 0.05 level, results will be reported by site. A *p*-value ≤ 0.025 will be used to determine if between group differences are statistically significant.

Because the patient populations at the three sites are different, we calculated a sample size of 106 per group for each site to provide adequate power to detect clinically important differences between groups at each site. The sample size estimates were obtained using a significance level of 0.025 to account for 2 primary outcome variables. The estimates of standard deviation (5.4 for the RMDQ and 2.0 for the NRS for average pain intensity over the past week) come from our pilot study [28]. This provides 80 % power to detect a 2.4 between group difference on the RMDQ and 92 % power to detect a 1.2 difference on the NRS. In the pilot study, there was 13 % and 11 % missing data in the UMC plus chiropractic care group at the week 2 and 4 assessment periods, respectively, but 39 % and 37 % in the UMC alone group. We increased the sample size to 125 per group at each site, assuming we would be able to keep our loss to follow-up at the week 6 endpoint at or below 15 %, due to the implementation of intensive follow-up procedures.

Secondary evaluations of the final models will compare group differences at week 12 to ascertain if the pattern seen at week 6 remains after the active care phase. Group differences will also be reported for week 2 and 4 to compare to the results of the pilot study. Secondary analyses will compare the percentage of patients with clinically meaningful improvement of at least 30 % relative to baseline at the week 6 endpoint on the co-primary outcome variables [49]. General estimating equations with a working covariance matrix will be used to estimate the differences in proportions between groups at each time point, with terms in the model for time (as a categorical variable), group, site, site-by-group, and time-by-group interactions, and the minimization variables. Consistent with the recent NIH Task Force recommendations [50] for a minimum dataset for chronic LBP, we will conduct an exploratory analysis over a range of improvement levels.

Two approaches to sensitivity analyses will be used to examine the possible effects of missing data on the results obtained from using all observed data for the co-primary outcome variables. Prior to conducting the sensitivity analyses, baseline variables that are predictive of missing outcomes will be identified with logistic regression models. The first approach will be under the assumption that data are missing at random and will use the Markov chain Monte Carlo method to impute missing values for each of the primary outcome variables based on the final mixed model covariates, the observed outcome variable at baseline and weeks 2, 3, 6, and 12, and the baseline variables predictive of missing data. The resulting datasets for each of 20 imputations will be analyzed with the linear mixed effects models that are fit with all observed data and the results will be combined. The second approach will be under the assumption that data are missing not at random. It will follow the pattern mixture approach described by Carpenter and Kenward [51] by first imputing missing values as described above for the missing at random approach and then for each participant in each treatment group for each imputation. The imputed observation will be decreased by different amounts representing different patterns of responses. The resulting datasets for each pattern will be analyzed and the estimates combined as described above. If results differ between the analysis of the observed data and that based on imputed full datasets under different missingness assumptions, multiple sets of results will be reported.

#### Secondary data analysis

The continuous secondary outcome variables will be analyzed with linear mixed effects regression as described above, but *p*-values ≤ 0.05 will be used to determine if between group differences are significant. The ordinal categorical variable representing the number of days that participants reported using medications for LBP over the past week will be analyzed over baseline and weeks 2, 3, 6, and 12 with a proportional odds model. Generalized estimating equations using all observed data with a working covariance structure will be used to fit the model.

### Protocol fidelity and quality assurance

#### Protocol fidelity

We are carefully tracking intervention and protocol adherence. Using the Armed Forces Health Longitudinal Technology Application, or patient electronic medical record, we are tracking all care received for LBP during the 3-month study duration. This includes both UMC visits, as well as chiropractic visits. Instances where participants who are allocated to receive UMC only but do receive chiropractic care during the 6 weeks of active care, as well as participants who are allocated to receive chiropractic care but do not will be classified as unanticipated events and documented in STaRS.

#### Internal quality assurance process

The lead PM conducts an internal quality assurance audit at each site on a quarterly basis for the purpose of maintaining data integrity, ensuring study protocol fidelity, and standardizing study operating procedures across all three sites. During the audit, the lead PM reviews regulatory documentation and informed consent documents. Electronic data are verified by comparing the paper source documents to the data entered in STaRS. Any errors discovered during the quarterly audits are documented, corrected by the site PM, and reported to the site PI, collaborating investigators, and appropriate regulatory bodies, if applicable.

During these site visits, the lead PM also meets with site PMs, PIs, DCs, and/or clinic command to facilitate communication about overall study status and discuss study timelines, as well as address site concerns or barriers interfering with study conduct. Information gathered during the site visits is conveyed to study co-investigators. In addition, the PCCR PI has a monthly conference call with study personnel at each clinical site to monitor study progress.

### Study event monitoring and reporting

#### Adverse events

We have defined an adverse event as any untoward medical occurrence presenting during the active study period (6 weeks) that may or may not have a causal relationship with study procedures [52]. A serious adverse event is defined as an event resulting in a condition considered as life-threatening, a congenital anomaly or birth defect, in-patient hospitalization, disability, permanent damage, death, or an occurrence that requires intervention to prevent death or significant disability. Adverse event information is being collected via 1) direct report to PM and/or 2) self-report during online assessments.

Participants are encouraged to contact the PM if there are any unplanned hospitalizations/procedures or for any other health-related events whether or not the participant considers them related to the study. When participants report adverse event information directly to a PM, the PM enters the adverse event information into STaRS, which generates an auto-notification message to the lead PM, designated trial clinician, and PI. Participants are also being prompted to answer questions about adverse events during the week 2, 4, and 6 online assessments. The lead PM and the central trial clinician review adverse event information received from online assessments on a weekly basis. The site PM will be asked to follow up with any participant who reports an adverse event to ascertain whether or not the event resulted in hospitalization or appeared to be an unexpected reaction or side effect from the study intervention. The lead PM facilitates the submission of any reportable adverse events to the respective IRBs, site medical monitor, and/or the Data and Safety Monitoring Committee (DSMC). Events not meeting the criteria for immediate reporting are submitted to the IRBs at the time of continuing review.

Each military study site has a medical monitor assigned to the study. The medical monitor is responsible for reviewing adverse events, as well as unanticipated events that may increase the risk to trial participants, any related serious adverse event, or related participant death. Events meeting these criteria are also submitted to the DSMC and the U.S. Army Medical Research and Material Command, Human Research Protection Office.

### Study limitations

Given the nature of this trial, it is not possible to blind either the treating clinicians or participants to treatment assignment. This is an important limitation to this study. However, all participants, clinicians, and study personnel are blinded to next treatment assignment, and all key study personnel and data analysts are blinded to group assignment. One could also argue that the pragmatic nature of this comparative effectiveness trial is a limitation given the resulting lack of homogeneity in treatment approach both within and across groups. The advantage of this approach is that the results are more applicable to “real world practice”; the disadvantage is that one must sacrifice the homogeneity inherent within an RCT.

We believe that a comparative effectiveness design is the best way to answer questions that will be meaningful to policy makers as they consider the appropriate role for chiropractic care in active duty military populations. Further, our experience in the conduct of clinical trials in MTFs has shown us that this type of trial is feasible to conduct in busy clinical practice settings. We will address this limitation by collecting detailed data on the treatments rendered to participants, for both analysis and reporting purposes.

## Discussion

Since LBP is one of the leading causes of disability among U.S. military personnel, it is important to find pragmatic and conservative treatments that will not only treat LBP, but could ultimately preserve low back function so that military readiness is maintained. In this trial, we will evaluate the effects of the addition of chiropractic care to UMC on LBP pain and disability. A pilot study compared chiropractic care plus standard medical care with standard medical care alone for active duty military personnel with acute LBP [28]. Improvements in pain and disability were significantly greater in the chiropractic care group. This comparative effectiveness study will evaluate whether these prior findings can be reproduced in a larger sample, across multiple sites, and with varied populations including individuals with subacute and chronic LBP. The information gleaned from this large, multisite trial may assist military healthcare providers to more effectively treat a highly prevalent condition responsible for high healthcare costs, debilitating effects on patients, and military readiness.

### Trial status

Recruitment began in September of 2012. As of November 20, 2015, 750/750 participants were allocated and recruitment was closed.

## Abbreviations

ACT: Assessment of Chiropractic Treatment
CRADA: Cooperative Research and Development Agreement
DC: Doctor of Chiropractic
DoD: Department of Defense
DSMC: Data and Safety Monitoring Committee
IRB: Institutional Review Board
LBP: low back pain
MTF: Military Treatment Facility
NHP: Naval Hospital Pensacola
NMCSD: Naval Medical Center San Diego
NRS: Numeric Rating Scale
PCCR: Palmer Center for Chiropractic Research
PI: Principal Investigator
PM: Project Manager
PRO: patient-reported outcome
PROMIS: Patient Reported Outcomes Measurement Information System
RCT: randomized controlled trial
RMDQ: Roland-Morris Disability Questionnaire
SQL: Structured Query Language
SSL: Secure Socket Layers
STaRS: Submission Tracking and Reporting System
UMC: usual medical care
WRNMMC: Walter Reed National Military Medical Center
